# Autoimmune encephalitis in children and adolescents

**DOI:** 10.1186/s42466-019-0047-8

**Published:** 2020-01-03

**Authors:** C. G. Bien, C. I. Bien

**Affiliations:** 1 Epilepsy Center Bethel, Krankenhaus Mara, Maraweg 17-21, 33617 Bielefeld, Germany; 2 Laboratory Krone, Bad Salzuflen, Germany

## Abstract

**Background:**

Autoimmune encephalitides with neural and glial antibodies have become an attractive field in neurology because the antibodies are syndrome-specific, explain the pathogenesis, indicate the likelihood of an underlying tumor, and often predict a good response to immunotherapy. The relevance and the management of antibody-associated encephalitides in the pediatric age group are to be discussed.

**Main body:**

Subacutely evolving, complex neuropsychiatric conditions that are otherwise unexplained should raise the suspicion of autoimmune encephalitis. Determination of autoantibodies is the key diagnostic step. It is recommended to study cerebrospinal fluid and serum in parallel to yield highest diagnostic sensitivity and specificity. The most frequently found antibodies are those against the N-methyl-D-asparate receptor, an antigen on the neural cell surface. The second most frequent antibody is directed against glutamic acid decarboxylase 65 kDa, an intracellular protein, often found in chronic conditions with questionable inflammatory activity. Immunotherapy is the mainstay of treatment in autoimmune encephalitides. Steroids, apheresis and intravenous immunoglobulin are first-line interventions. Rituximab or cyclophosphamide are given as second-line treatments. Patients with surface antibodies usually respond well to immunotherapy whereas cases with antibodies against intracellular antigens most often do not.

**Conclusion:**

With few exceptions, the experience in adult patients with autoimmune encephalitides can be applied to patients in the pediatric age range.

## Background

The discovery of immunoglobulin G (IgG) antibodies against proteins on nerve cell surfaces has been perceived as a major advance and even a breakthrough in neurology. The first specific antibodies that have relevance and validity until to date are those against the N-methyl-D-aspartate receptor (NMDAR) [1]. These were followed by those against leucine-rich glioma inactivated protein 1 (LGI1) [2, 3], contactin-associated protein-2 (CASPR2) [3, 4] and others. These new “surface antibodies” are diagnosed by incubating diluted serum or undiluted (or mildly diluted) cerebrospinal fluid (CSF) with human embryonic kidney (HEK) cells transfected with the antigens of interest [5]. The binding of antibodies is visualized by a secondary anti-human IgG antibody coupled to a dye that can be visualized under the microscope.

The combination of four properties makes these autoantibodies so valuable:

- their syndrome specificity,
- their pathogenetic explanatory power,
- the frequently good treatability of the associated autoimmune central nervous system (CNS) syndromes
- the indication of the underlying tumor probability (paraneoplastic syndromes).

Initially, these antibodies were found in adults. It quickly became clear that they also occur in childhood and are important there [6]. For all age groups, it has been shown in recent years that the antibody detection methods have a good sensitivity. Diagnostic problems are mainly related to specificity of antibody diagnostics. For example, the specificity of antibodies of immunoglobulin classes IgA and IgM for neurological syndromes is doubtful. Currently, no diagnoses should be based on the detection of IgA or IgM antibodies [7]. Another issue are minimum antibody titers for meaningful diagnoses. For CASPR2 IgG antibodies, only antibodies at a minimum serum titer level of 1:128 (or antibodies at any titer in the CSF) are specific for the diagnosis of autoimmune encephalitis [8]. The validation and interpretation of positive antibody findings based on clinical presentation is of great importance in order to avoid false-positive findings.

The list of potentially relevant antibodies that is known today may not yet be complete, as the field is still young and there are still reports of new associations from the neuropediatric age range [5]. Recently published clinical diagnostic criteria for autoimmune encephalitides [9] are of great help in identifying candidates for antibody testing and in checking the relevance of an antibody finding. The criteria should, as the authors of the Position paper say in the section “General scope and objectives” say, be used with caution in patients < 5 years of age because clinical presentation may be different in this age range [9].

In addition to autoimmune encephalitis, which primarily affects the gray matter of the CNS, demyelinating diseases can also be elucidated with the help of autoantibodies. Children and adolescents presenting with acute disseminated encephalomyelitis (ADEM), myelitis or optic neuritis often harbor antibodies to the myelin oligodendrocytic glycoprotein (MOG). The most sensitive and specific method to detect these antibodies is the use of live MOG-transfected HEK cells. Such a live cell assay is superior to a commercial fixed cell assay [10]. MOG antibodies predict a good response to immunotherapy [11].

The existing case series show the correlations between syndromic presentations and antibodies given in the upper part of Table 1. For a review of autoimmune encephalitides in the pediatric age range, see [12].

**Table 1:** Features and antibodies characteristic of autoimmune encephalitis in children and adolescents. The list of antibodies may not be exhaustive, as the field is still young and there are still reports of new associations from the pediatric age range. Abbreviations are spelled out in the list at the beginning of the article

Features	Typical antibodies in the pediatric age range: Anti-…
Syndromes	
Limbic encephalitis	LGI1, CASPR2, GABABR, GAD65, Hu, DNER
Encephalopathy (in the sense of a diffuse affection of brain function)	NMDAR, GABABR, mGluR5
Brainstem encephalitis	GQ1b
Cerebellitis/autoimmune cerebellar syndrome	DNER, VGCC, NMDAR, mGluR1^a^
Opsoclonus myoclonus syndrome	Hu, GAD65
Progressive encephalomyelitis with rigidity and myoclonus	GAD65, GlyR
Demyelinating diseases	MOG, Aquaporin-4, NMDAR*
Patient and medical history	
Girls	GAD65 (NMDAR?)
Other autoimmune disease	GAD65
Epilepsy onset along with prominent psychiatric or cognitive symptoms	LGI1, CASPR2, NMDAR, GABABR, GAD65, Hu, DNER
Onset with status epilepticus or very high seizure frequency	NMDAR, LGI1, GABAAR, GABABR, GAD65
Non-viral secondary disease after viral encephalitis	NMDAR and not further characterized surface antigens
Encephalopathy after respiratory infection	MOG
Neoplasm	Onconeural; mGluR5 (Hodgkin disease)
Paraclinical findings	
EEG: “extreme delta brush”	NMDAR
Increased CSF count or autochthonous oligoclonal bands	All except LGI1
MRI: encephalitic lesion(s)	LGI1, CASPR2, GABABR, GAD, Hu, DNER
MRI: demyelination	MOG, Aquaporin-4, NMDAR
Encephalitic histopathology	All

^a^Unpublished own observation

*Overlap syndromes

In the following, present knowledge and recent challenges in the diagnostic and therapeutic management of pediatric patients with (suspected) autoimmune encephalitis are discussed:

- Which patients should be evaluated regarding a potential autoimmune encephalitis?
- How to do an appropriate work-up of such patients? How frequent are which antibodies in the pediatric population?
- How to treat patients with the diagnosis of autoimmune encephalitis?

### Patients suspicious for autoimmune encephalitis

A key feature of autoimmune encephalitides are the subacute evolution of otherwise uncommon combinations of neurological, cognitive and psychiatric symptoms. The most important paraclinical features are encephalitic magnetic resonance imaging (MRI) lesions or an inflammatory CSF. The “Graus criteria” [9] make use of these features in their introductory category of “possible autoimmune encephalitis” (Table 2). This useful definition developed within adult neurology can usually be applicated to adolescents but is not always fully adequate in children < 5 years of age, because their presentation may differ: Some young cases present without the full spectrum of symptoms (so that the aspect of the above-mentioned “uncommon symptom *combination*” is not evident); in other instances, the dominant features are not observed to adults (e.g. the massive arterial hypertension in young children with Morvan syndrome and CASPR2 antibodies [13]). It therefore remains a challenge to clinical experience whom to further evaluate for suspected autoimmune encephalitis.

**Table 2:** Criteria for “possible autoimmune encephalitis” (simplified according to Panel 1 in [25])

All three must be fulfilled:	
1	Subacute onset (< 3 months) of working memory deficits (short-term memory loss), altered mental status (decreased or altered level of consciousness, lethargy or personality change) or psychiatric symptoms
2	≥1 of the following: - New focal CNS findings - Seizures not explained by a previously known seizure disorder - CSF pleocytosis (white blood cell count > 5/μl) - MRI features suggestive of encephalitis
3	Reasonable exclusion of alternative causes^a^

^a^CNS infections, septic encephalopathy, metabolic encephalopathy, drug toxicity (Including use of illicit drugs, direct neurotoxic effect of prescribed drugs or through induction of seizures, posterior reversible encephalopathy, idiosyncratic reaction [e.g. neuroleptic malignant syndrome], drug interaction [e.g. serotoninergic syndrome] or drug withdrawal), cerebrovascular disease, neoplastic disorders, Creutzfeldt-Jakob disease, epileptic disorders, rheumatologic disorders (e.g., lupus, sarcoidosis, other), Kleine-Levin syndrome, Reye syndrome (children), mitochondrial diseases, inborn errors of metabolism (children)

### Work-up of children and adolescents with suspected autoimmune encephalitis

MRI and CSF diagnostics are indispensable in children and adolescents with suspected autoimmune encephalitis. On the one side, they help identifying differential diagnoses (e.g., infectious encephalitides), on the other hand, they may strengthen the suspicion (e.g., by demonstrating the typical bilateral mediotemporal T2/FLAIR signal increase of limbic encephalitis or identifying autoantibodies in CSF, which is crucial for NMDAR antibodies). The central diagnostic step is the test for neural antibodies. In most labs, this is today done by means of a panel diagnostic. “Biochips” containing several fields with differently transfected HEK cells permit testing for a broad range of surface antibody reactivities in one run [14]. In addition, immunoblots containing onconeural antigens are applied. The reason is that pediatric patients may occasionally harbor onconeural antibodies, e.g. Hu antibodies in the presence of a neuroblastoma [15]. Ideally, a tissue-based assay (usually, a section of rodent brain) is applied in parallel to identify less common or potentially novel antibodies against surface antigens by a neuropil staining [5].

A frequent question is if a serum, a CSF or a serum-CSF-pair investigation is recommended. Reasons for potentially restricting the test materials are the distress for the patients by a lumbar puncture and the lower costs if only one instead of two materials is studied. In recent years, international authorities from different institutions have uniformly recommended the simultaneous testing of CSF and serum in cases of suspected autoimmune encephalitis, see e.g. [16]. The key reason is that in some cases, the antibodies are detectable only in one of the two materials. For example, NMDAR antibodies are not always found in serum [17]; in contrast, LGI1 or MOG antibodies are not always found in the CSF [16, 18]. Exceptions to the general recmmendation to test CSF-serum pairs may be girls with encephalopathy suggesting anti-NMDAR encephalitis or post-herpes autoimmune encephalitis. Both can often be finally diagnosed by testing for NMDAR antibodies in CSF only [19] so that CSF may be sufficient. However, the clinical presentation is often ambiguous, or a patient may have other antibodies in addition to NMDAR antibodies. An example are antibodies against MOG in the case of overlaps of anti-NMDAR encephalitis with demyelinating disorders [20]. Some patients with an encephalitis defined by antibodies against the γ-aminobutyric acid-A receptor (GABAAR) may look like patients with anti-NMDAR encephalitis. They would be detected at best with a delay if initially only an NMDAR antibody test was done. In this situation, the correct antibody would be only identified by using a biochip together with a tissue-based assay. The reason is that GABAAR transfected HEK cells are not yet regularly available as part of the biochips but can be readily suspected on the tissue-based assay and confirmed in a research laboratory.

**Fig. 1:**
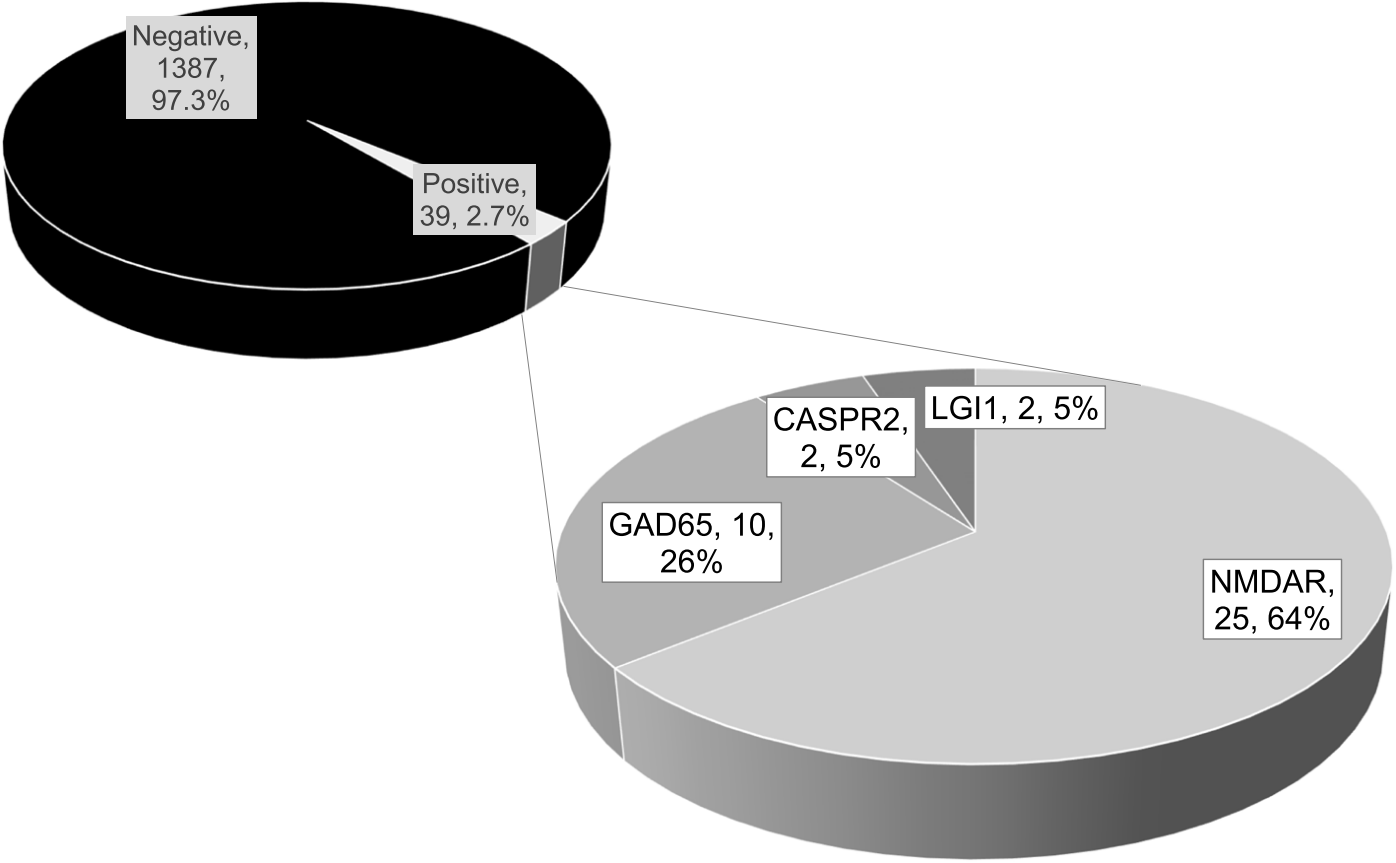
Frequency of positive results from the testing of 1426 patients < 18 years in the years 2011–2015 in the antibody laboratory of the Epilepsy Center Bethel. For each patient, only the earliest sample(s) were included. Absolute numbers and percentages are indicated in the labels

**Fig. 2:**
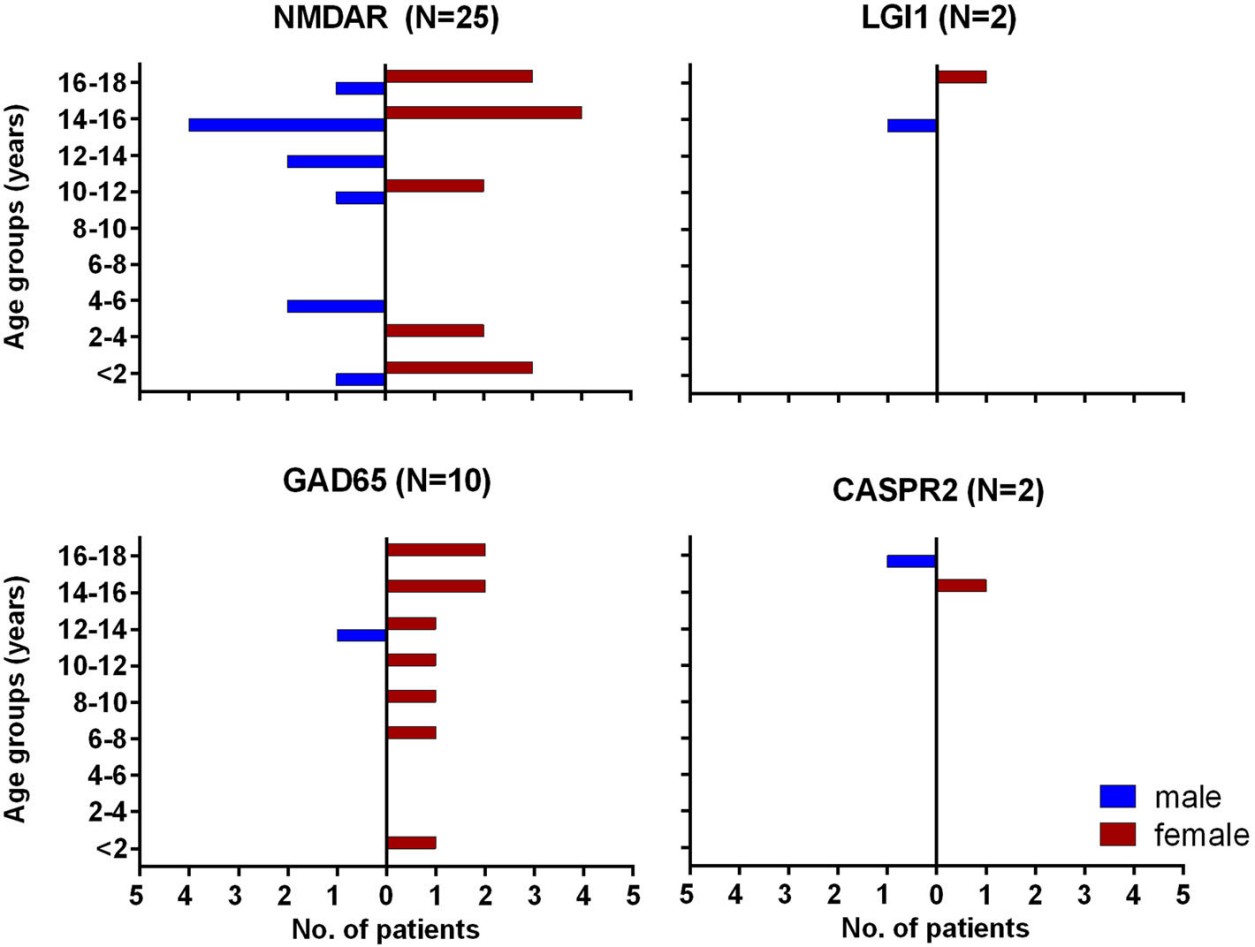
Age and sex distribution of antibody-positive patients. Males: blue; females: red. The patients with GAD65 antibodies are predominantly female, whereas in the other groups, the relationship is equal, even with NMDAR antibodies (56% female). Only one girl (4% of all patients with NMDAR antibodies) had paraneoplastic disease with an ovarian teratoma. The figures in a recent Chinese pediatric study were: 61% females (*N* = 54), one case with ovarian teratoma (1.1%) [28]. One pediatric series from the US (*N* = 32) had different results: The authors found 81% female patients and 25% paraneoplastic cases [25]. One reason seems to be that African-American patients particularly frequently have paraneoplastic anti-NMDAR encephalitis [26]

The frequency of positive results during routine diagnostics in the antibody laboratory in the Epilepsy Center Bethel using a broad panel for neural antibodies from 2011 to 2015 is shown in Fig. 1. Figure 2 depicts the age distribution of the four most common antibodies. An overview (against the NMDAR, LGI1, CASPR2, the AMPAR1/2, the GABABR, the GlyR, GAD65, Hu, Yo, Ri, CV2, amphiphysin, Ma2, and Sox1) over antibodies, and associated syndromes can be found in Table 3.

**Table 3:** Antibodies and encephalitic syndromes in children and adolescents

Antibodies against	Syndromes	Further reading
NMDAR	Encephalopathy	[25, 28]
GAD65	Limbic (and extralimbic) encephalitis, stiff-man-syndrome (SMS)/progressive encephalomyelitis with rigidity and myoclonus (PERM)	[41–44]
LGI1	Mainly limbic encephalitis	[34, 35]
CASPR2	Mainly diffuse encephalitis, encephalopathy or seizure disorder	[34]
LGI1 and CASPR2 (double positive)	Mainly Morvan syndrome or neuromyotonia	[34]
GlyR	SMS/PERM^a^	[45]
γ-aminobutyric acid-A receptor (GABAAR)	Encephalitis	[46–48]
γ-aminobutyric acid-B receptor (GABABR)	Encephalitis with opsoclonus, ataxia, chorea, and seizures	[49]
Onconeural antigens (Hu and others)	Mostly paraneoplastic limbic encephalitis	[41, 50–52]
GQ1b	Bickerstaff brainstem encephalitis	[40]
mGluR1	Cerebellitis	Own unpublished observation
mGluR5	Encephalitis with cognitive and psychiatric problems, seizures	[53]
Basal ganglia (dopamine receptor 2 [DR2])^b^	Basal ganglia encephalitis, chorea minor Sydenham, Tourette’s syndrome	[54–56]

^a^GlyR with syndromes different from SMS/PERM are probably non-specific [8]

^b^These antibodies were originally described in 12 pediatric patients with basal ganglia encephalitis or Sydenham chorea (chorea minor), occasionally Tourette’s syndrome (not, however, in Pediatric Autoimmune Neuropsychiatric Disorders Associated with Streptococcal Infections [PANDAS]) [54]. Until now, these results have not been reproduced by other laboratories

An additional note is required for MOG antibodies. Two aspects need to be considered here: These antibodies are not reliably detected by fixed cells and require live cell assays for optimal results [10]; therefore, any biochip technique comprising MOG cells would be suboptimal for their detection. Second, MOG antibodies have been traditionally linked with demyelinating disease; there are, however, encephalitic presentations with seizures and neocortical or basal ganglia lesions but no white matter demyelination with high-titer MOG antibodies in adults [21, 22]. It is conceivable that such cases have been underrecognized in the pediatric population so far. One may tentatively suggest MOG antibody serum testing through a live cell assay in cases with one or more seizures plus neocortical or basal ganglia lesions and negative results with neural autoantibodies on the usual biochip panel.

### Treatment of children and adolescents with autoimmune encephalitis

Most data exist on the treatment of patients with NMDAR encephalitis. Internationally, there is no difference in the therapeutic approach to children/adolescents and adults [23]. The usual scheme is that of a first-line and a second-line immunotherapy approach as detailed in Table 4. This concept was derived from the retrospective analysis of 105 patients with that disease [24]. In addition to immunotherapy, symptomatic treatments against seizures, agitation, autonomic problems and so on are regularly given; > 40% of patients require intensive care [25–27]. Underlying tumors (mostly ovarian teratomata) need to be removed in addition to immunotherapy. The outcome of anti-NMDAR encephalitis is impressively good: 81% live independently 2 years after diagnosis [26]. Very similar results emerged from a pediatric cohort: 84% of patients were said to have had “complete recovery” in a Chinese series [28]. An important observation is that earlier treatment and earlier escalation to second-line treatments are associated with better outcome in children [29]. Authorities recommend escalation to second-line therapy after 10–14 days without significant improvement upon first-line therapy, especially, if the patients are on the intensive care unit [24]. Relapses are not uncommon. The relapse rate in children followed up for 1–5 years was 13.5% [28]. Relapses are usually milder than the initial disease episode and respond even faster to immunological treatment [30].

**Table 4:** First-line and second-line therapy in autoimmune encephalitides according to [24]

First line	
Corticosteroids	No details on doses or durations given
Plasma exchange	
Intravenous immunoglobulins	
Second line (if first line did not work within 10 days^a^)	
Rituximab	4 × 375 mg/m^2^ i.v., weekly administration
Cyclophosphamide^b^	750 mg/m^2^ i.v. per month

^a^This has been said for anti-NMDAR encephalitis. In other, less severe forms of autoimmune encephalitis, one may wait longer before one moves on to second-line therapy. ^b^In pediatric patients, cyclophosphamide is less frequently used compared to rituximab

Recent animal data suggest that NMDAR antibodies in pregnant mothers may cross the placenta and may cause neuropsychiatric disorders in their offspring [31].

From anti-NMDAR encephalitis, the first-line/second-line approach has been extended to the encephalitides with other antibodies [32]. Escalation usually does not need to be done as rapidly as with NMDAR antibodies. It has been recommended to observe the effect for 1 to 2 months before moving on to a second-line intervention [33]. Patients with LGI1 or CASPR2 antibodies usually respond well to immunotherapy [28, 34, 35].

In contrast, antibodies against intracellular antigens – most frequently against glutamic acid decarboxylase 65 kDa (GAD65), rarely against an onconeural antigen -, portend a less favorable outcome. Usually, these patients take a chronic course. The most common scenario is the development of pharmacoresistant focal epilepsy. Fortunately, in many cases, the patients do not chronically deteriorate [36]. There are almost no reports on successful immunological therapies in patients with GAD65 antibodies [37].

An open question is how long one should wait for the effect of full first-line and second-line therapy. A large retrospective series showed an increasing number of improving patients over 2 years. This was the end of the observational period [26]. Later improvements are therefore possible. Recently, agents like tocilizumab or bortezomib have been advocated as third-line therapies, especially for severe anti-NMDAR encephalitis. Support for this comes from case studies [38, 39]. It remains open to discussion how big the influence of those novel agents in the immunological polypharmacy was.

For Bickerstaff encephalitis, a recent review found in the published data a good response to the predominant application of intravenous immunoglobulins (IVIG) followed by steroids and plasma exchange if needed [40]. With MOG-antibodies, therapy is based on better studied conditions like neuromyelitis optica spectrum disease with aquaporin-4 antibodies. One uses corticosteroids, IVIG, immunosuppressants (like mycophenolate mofetil, azathioprine or methotrexate) and rituximab. All these interventions seem associated with a reduction in relapse rate [16].

## Summary

Most neuropediatric cases of autoimmune encephalitis harbor NMDAR antibodies in CSF. Cases with other antibodies occur. The frequency of MOG antibodies in pediatric encephalitides remains to be determined; it might be higher than known today. The existing data justify the use of multiparametric testing for neural antibodies at disease onset in serum and CSF with panel diagnostic through biochips. There are no fundamental differences between autoimmune encephalitides in pediatric and adult cases and their management. Neuropediatricians can therefore rely on insights from adult neurology. Therapy follows a first-line (steroids, IVIG, apheresis) and second-line (rituximab, less frequently cyclophosphamide) concept.

## Data Availability

Not applicable.

## Abbreviations

ADEM: Acute disseminated encephalomyelitis myelitis
CASPR2: Contactin-associated protein-2
CNS: Central nervous system
CSF: Cerebrospinal fluid
DNER: Delta/Notch-like EGF-related receptor
DR2: Dopamine receptor 2
GABAAR: γ-aminobutyric acid-A receptor
GABABR: γ-aminobutyric acid-B receptor
GAD65: Glutamic acid decarboxylase 65 kDa
GlyR: Glycine receptor
HEK cells: Human embryonic kidney cells
IgA, IgG, IgM: Immunoglobulin A, G, M
IVIG: Intravenous immunoglobulins
LGI1: Leucine-rich glioma inactivated protein 1
mGluR: metabotropic glutamate receptor 1
mGluR5: metabotropic glutamate receptor 5
MOG: Myelin oligodendrocytic glycoprotein
MRI: Magnetic resonance imaging
NMDAR: N-methyl-D-aspartate receptor
